# Loss of microRNA-23–27–24 clusters in skeletal muscle is not influential in skeletal muscle development and exercise-induced muscle adaptation

**DOI:** 10.1038/s41598-018-37765-3

**Published:** 2019-01-31

**Authors:** Minjung Lee, Shogo Wada, Satoshi Oikawa, Katsuhiko Suzuki, Takashi Ushida, Takayuki Akimoto

**Affiliations:** 1 Division of Regenerative Medical Engineering, Center for Disease Biology and Integrative Medicine, Graduate School of Medicine, The University of Tokyo, Tokyo, 113-0033 Japan; 2 Graduate School of Comprehensive Human Science, University of Tsukuba, Ibaraki, 305-8571 Japan; 3 Faculty of Sport Sciences, Waseda University, Saitama, 359-1192 Japan; 4 Present Address: Department of Medicine and Cardiovascular Institute, Perelman School of Medicine, University of Pennsylvania, Philadelphia, PA USA

## Abstract

MicroRNAs are small regulatory noncoding RNAs that repress gene expression at the posttranscriptional level. Previous studies have reported that the expression of miR-23, miR-27, and miR-24, driven from two miR-23–27–24 clusters, is altered by various pathophysiological conditions. However, their functions in skeletal muscle have not been clarified. To define the roles of the miR-23–27–24 clusters in skeletal muscle, we generated double-knockout (dKO) mice muscle-specifically lacking the miR-23–27–24 clusters. The dKO mice were viable and showed normal growth. The contractile and metabolic features of the muscles, represented by the expression of the myosin heavy chain and the oxidative markers PGC1-α and COX IV, were not altered in the dKO mice compared with wild-type mice. The dKO mice showed increased cross-sectional areas of the oxidative fibers. However, this dKO did not induce functional changes in the muscles. The dKO mice also showed normal adaptation to voluntary wheel running for 4 weeks, including the glycolytic-to-oxidative fiber type switch, and increases in mitochondrial markers, succinate dehydrogenase activity, and angiogenesis. In conclusion, our data demonstrate that the miR-23–27–24 clusters have subtle effects on skeletal muscle development and endurance-exercise-induced muscle adaptation.

## Introduction

MicroRNAs (miRNAs) are short noncoding RNAs that negatively regulate gene expression at the posttranscriptional level^1^. This repressive regulation predominantly relies on the seed regions in the 5′ regions of the miRNAs, which bind to their complementary sequences, usually in the 3′ untranslated regions (UTRs) of the target mRNAs^2^. A single miRNA has hundreds of mRNA targets and a single mRNA is targeted by multiple miRNAs^2^. Because a miRNA modestly represses the expression of a number of its target genes^3,4^ and most human mRNAs are predicted to be conserved targets of miRNAs^5^, miRNAs are considered to be critical regulatory molecules that fine tune global gene expression.

The capacity of miRNAs to repress their target mRNAs largely depends on their expression levels^6,7^. Therefore, miRNAs highly expressed in a specific tissue may have significant effects on gene expression in that tissue. For example, several miRNAs, including miR-1, miR-133, and miR-206, have been identified as specifically and highly expressed in striated muscle^8^ and their functions have been extensively studied. A number of loss-of-function studies have reported that the conventional knockout of miR-1 and miR-133a impaired heart development, causing neonatal and embryonic lethality^9–14^, although less than 25% of miR-133a-1/miR-133a-2 double KO (miR-133a dKO) mice survived until adulthood, with dilated cardiomyopathy^10^. The surviving miR-133a dKO mice displayed abnormalities in their skeletal muscle after 4 weeks of age, characterized by progressive centronuclear myopathy in the fast-twitch myofibers, mitochondrial dysfunction, and a glycolytic-to-oxidative muscle type switch^11^. Furthermore, at 3 months of age, the miR-133a dKO mice displayed a reduced capacity for endurance exercise and lower mitochondrial biogenesis after 6 weeks of treadmill exercise^11,15^.

We have previously demonstrated that miRNAs produced from the miR-23–27–24 clusters are also highly expressed in skeletal muscle^16^. There are two paralogous miR-23–27–24 clusters: miR-23a–27a–24-2 (miR-23a cluster) and miR-23b–27b–24-1 (miR-23b cluster) located on chromosomes 8 and 13, respectively, in the mouse genome and on chromosomes 19 and 9, respectively, in the human genome. A number of studies have reported that the miRNAs in the miR-23–27–24 clusters are altered in response to physiological and/or pathological changes in the skeletal muscle. A recent study reported that miR-24 is downregulated in response to acute contusion muscle injury^17^, and other studies have reported that muscle wasting conditions, such as diabetes and limb immobilization, are associated with the downregulation of miR-23 and miR-27 in the skeletal muscle^18–20^. Numerous studies have reported that miRNA expression in skeletal muscle is also altered by various types of exercise^21^, and have suggested that these changes in miRNAs contribute to the beneficial effects of exercise^22,23^. It has been reported that miR-23 is downregulated in the skeletal muscle by a single bout of acute endurance exercise, in both mice and humans^24,25^.

Because the miR-23–27–24 clusters are highly expressed in skeletal muscle and their expression is associated with many pathophysiological conditions, we speculated that the miR-23–27–24 clusters play important roles in the skeletal muscle biology. However, the functions of the miR-23–27–24 clusters in the skeletal muscle remain unclear. In this study, to investigate their functions, we generated mice in which the miR-23–27–24 clusters were muscle-specifically knocked out and determined their muscle phenotypes. We also examined the changes in the skeletal muscle phenotypes of the knockout mice in response to endurance exercise. This is the first study to report the muscle-specific loss of function of the miR-23–27–24 clusters *in vivo*.

## Results

### miRNA expression in the muscle-specific miR-23a/b-cluster-knockout mice

**Figure 1:**
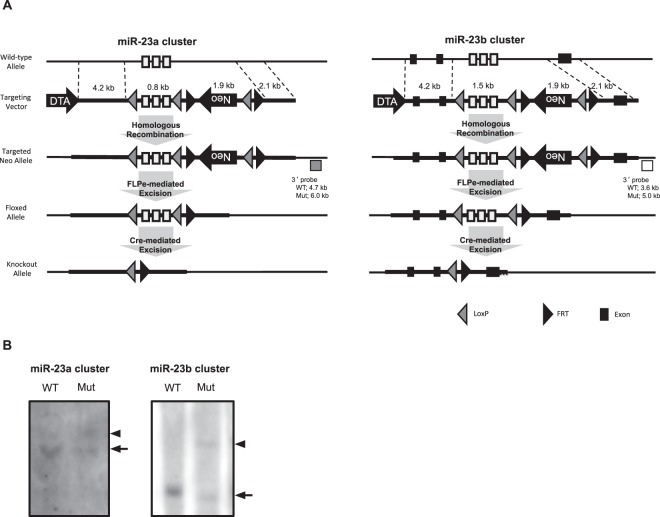
Schematic representation of the targeted deletion of miR-23–27–24 clusters. (**A**) Targeted deletion of miR-23a and miR-23b clusters. (**B**) Representative images of Southern blotting analysis with 3′ external probes specific for the WT (arrows) and mutant alleles (arrowheads) of miR-23a and miR-23b clusters.

**Figure 2:**
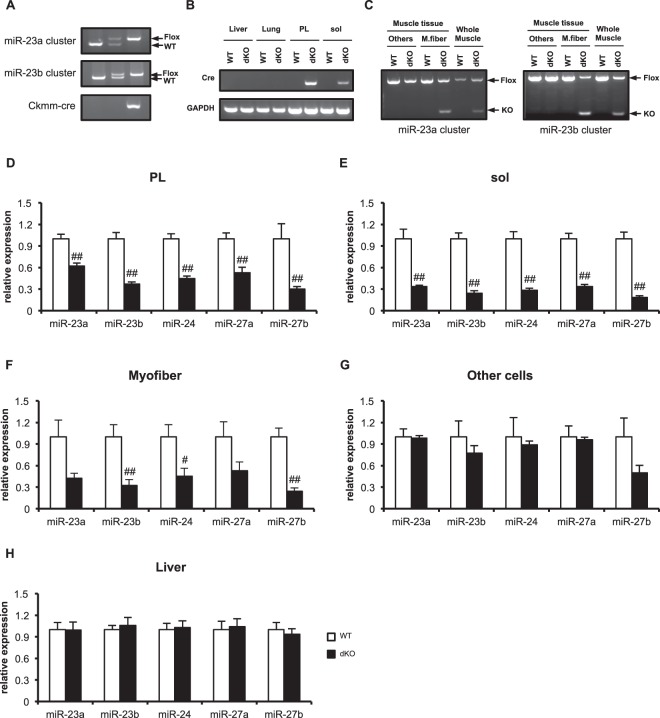
Muscle-specific knockout of miR-23–27–24 clusters. (**A**) Representative images of the genotyping of the floxed and WT alleles of miR-23–27–24 clusters and the Ckmm-cre allele from mouse genomic DNA. (**B**) mRNA expression of Cre in liver, lung, plantaris (PL) and soleus (sol) of the WT and dKO mice. (**C**) PCR with genomic DNA to detect Cre-induced recombination in skeletal muscle. Other cells include myoblasts and fibroblasts, etc., M. fiber: isolated myofibers, Whole muscle: whole EDL muscle, Flox: floxed allele. KO: knockout allele. (**D**–**H**) Expression of mature miRNAs generated from miR-23–27–24 clusters; miR-23a, miR-23b, miR-24, miR-27a, and miR-27b in plantaris muscle (**D**) soleus muscle (**E**) myofibers (**F**) other cells in muscle tissue (**G**) and liver (**H**) of WT and dKO mice. Data are means ± SEM (n = 5–6). ^##^P < 0.01 compared with WT.

To generate the muscle-specific-miR-23a/b-cluster KO (dKO) mice, we produced miR-23a/b-cluster-floxed mice (Fig. 1) and cross-bred the miR-23a/b-cluster-floxed mice with Ckmm-cre mice (Fig. 2A). The dKO mice were born at the normal Mendelian rate, with no abnormalities in their appearance. The body, heart, and muscle weights did not differ between the dKO mice and their littermate controls (wild type) (Table 1). To confirm the Ckmm-cre (also called Mck-cre)-induced recombination, we first determined expression of Cre recombinase in muscle and non-muscle tissues. The Cre mRNA was detected in the dKO mouse skeletal muscle (both in plantaris (PL) and soleus (sol)), but not in non-muscle tissues (Fig. 2B). Since the Ckmm promoter is activated in fully differentiated muscle fibers^26^, we examined the degree of the Ckmm-cre-induced recombination in isolated myofibers from adult skeletal muscle. We observed that the recombination was occurred only in myofibers but not in other cells in muscle tissue of the dKO mice (Fig. 2C).

**Table 1:** Bodyweight, heart and skeletal muscle weight.

Genotype	Body weight (g)	Heart (%)	PL (%)	Sol (%)
WT	26.01 ± 0.47	0.430 ± 0.008	0.062 ± 0.001	0.032 ± 0.001
dKO	26.89 ± 0.52	0.428 ± 0.007	0.064 ± 0.001	0.032 ± 0.001

WT, control littermate mice. dKO, Ckmm-cre induced muscle-specific miR-23a/b clusters double-KO mice. Heart (n = 8 for each group), plantaris (n = 6 for each group), and soleus (n = 6 for each group) weights (n = 7 for each group) per bodyweight were presented as percentage values. Data are presented as means ± SEM.

We next quantified the mature miRNA expression from the miR-23–27–24 clusters in the dKO mice skeletal muscles. These mature miRNAs were all significantly reduced in the plantaris (Fig. 2D) and soleus muscles (Fig. 2E) of the dKO mice compared with their levels in the control mice. We also confirmed that the mature miRNAs were decreased only in myofibers (Fig. 2F) but not in other cells of muscle tissue (Fig. 2G) such as myoblasts and fibroblasts of the dKO mice. To confirm that the deletion of the miRNAs was muscle specific, we quantified the expression of these miRNAs in the liver. The expression of the mature miRNAs from the miR-23–27–24 clusters in the liver was not altered by cross-breeding with the Ckmm-cre mice (Fig. 2H).

### Skeletal muscle phenotype of the muscle-specific miR-23–27–24-cluster-knockout mice

Skeletal muscle is a complex of muscle fibers that express distinct contractile proteins. Rodent skeletal muscle fibers express four different subtypes of MyHC: type I (slow-twitch and oxidative), type IIa (fast-twitch and oxidative), type IId/x (fast-twitch and glycolytic), and type IIb (fast-twitch and glycolytic). Because the combination of these fiber types determines the contractile and metabolic features of the whole muscle tissue, we first determined the expression of the MyHC isoforms and oxidative markers in the skeletal muscle. Two different muscles with different compositions of the MyHC types were analyzed: the soleus, which is mainly composed of oxidative type I and IIa fibers, and the plantaris, which is mainly composed of glycolytic type IIa and IIb fibers.

**Figure 3:**
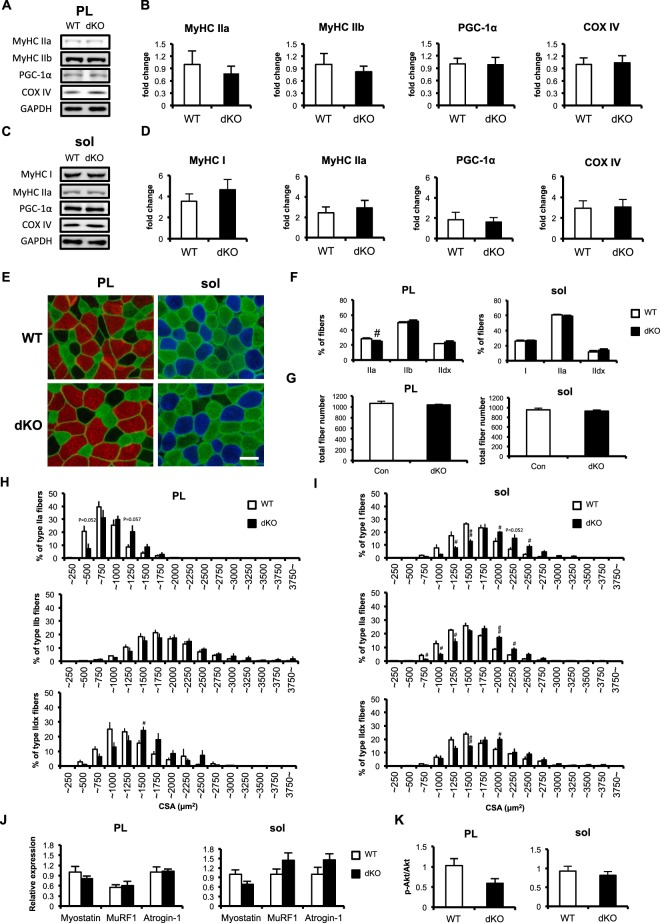
Skeletal muscle phenotype of miR-23–27–24-cluster-dKO mice. (**A**) Representative images of western blotting analysis of plantaris muscle. (**B**) Quantitative data for the protein expression of MyHC type IIa, MyHC type IIb, PGC-1α, and COX IV. Data are means ± SEM (n = 6). (**C**) Representative images of western blotting analysis of soleus muscle. (**D**) Quantitative data for the protein expression of MyHC type I, MyHC type IIa, PGC-1α, and COX IV. Data are means ± SEM (n = 5–6). (**E**) Representative images of plantaris and soleus muscles immunofluorescently stained for MyHC type IIa fibers and dystrophin in green, and type IIb fibers in red. MyHC type IId/x fibers are unstained (black). Scale bar, 50 μm. (**F**) Proportions of muscle fiber types in plantaris and soleus muscles. Proportion of each fiber type is presented as a percentage. Muscle fibers in entire cross-sections were counted. I, MyHC type I; IIa, MyHC type IIa; IIb, MyHC type IIb; IId/x, MyHC type IId/x. Data are means ± SEM (n = 4–5). ^#^P < 0.05 compared with WT. (**G**) Total fiber numbers in plantaris and soleus muscles. Muscle fibers in entire cross-sections were counted. Data are means ± SEM (n = 4–5). (**H**) Distributions of MyHC type IIa fibers (top) MyHC type IIb fibers (middle), and MyHC type IId/x fibers (bottom) in plantaris muscle. Graphs show percentages of fibers per cross-sectional area (CSA). Data are means ± SEM (n = 5). (**I**) CSA distributions of MyHC type I fibers (top), MyHC type IIa (middle), and MyHC type IId/x fibers (bottom) in soleus muscle. Data are means ± SEM (n = 5). (**J**) mRNA expression of Myostatin, MurF1 and Atrogin-1 in WT and dKO mice skeletal muscle. Data are means ± SEM (n = 6). K, Quantitative data for the phospho-Akt/Akt by western blotting in WT and dKO mice skeletal muscle. Data are means ± SEM (n = 6).

The expression of MyHC IIa and IIb proteins in the plantaris muscle was not altered by the muscle-specific dKO of the miR-23–27–24 clusters (Fig. 3A,B). The expression of PGC-1α, a master regulator of mitochondrial biogenesis, and COX IV, an enzyme complex involved in the mitochondrial respiratory electron transfer chain, was also unchanged by the dKO. Consistent with the observations in the fast plantaris muscle, there was no significant difference between the dKO and WT mice in the protein expression of MyHC I or IIa, PGC-1α, or COX IV in the slow soleus muscle.

To further understand the effects of the miR-23–27–24 clusters on the contractile properties of the skeletal muscle, we determined fiber-type compositions and cross sectional areas (CSAs) of the plantaris and soleus muscles (Fig. 3E). There were no differences in the muscle-fiber compositions of the plantaris or soleus muscles between the dKO and WT mice, except that the proportion of MyHC IIa fibers was reduced in the dKO plantaris muscle (Fig. 3F). The numbers of muscle fibers in both the plantaris and soleus were similar in the WT and dKO mice (Fig. 3G). The CSA analysis of each MyHC fiber type revealed that the CSAs of the type IIa and IId/x fibers were larger in the plantaris muscles of the dKO mice than in the WT, whereas the CSA of the type IIb fiber was not altered (Fig. 3H). The CSAs of the type I, IIa, and IId/x fibers in the soleus muscles of the dKO mice were also larger than those in the WT (Fig. 3I). We then examined expressions of muscle hypertrophy and atrophy-related genes including MuRF1, Atrogin1 and Myostatin that are reported to be targets of miR-23a/b cluster miRNAs^16,27^. We observed that these mRNAs were not changed in the dKO mice (Fig. 3J). We also examined whether the signaling pathway for protein synthesis during muscle hypertrophy^28^ was increased in the dKO mice and observed that phosphorylated Akt (p-Akt) was not changed in the dKO mice (Fig. 3K). Indeed, there was no significant difference in the muscle strengths of the WT and dKO mice (Table 2), suggesting that the increased CSAs of the muscle fibers in the dKO mice did not influence their muscle strength.

**Table 2:** Grip test.

Genotype	Strength (N/g)
WT	0.06 ± 0.007
dKO	0.07 ± 0.003

Grip strength per bodyweight (N/g). WT (n = 3), control littermate mice. dKO (n = 3), Ckmm-cre induced muscle-specific miR-23a/b clusters double-KO mice. Data are presented as means ± SEM.

### miR-23–27–24 clusters are not essential for the adaptation of adult skeletal muscle to 4 weeks of voluntary running

**Figure 4:**
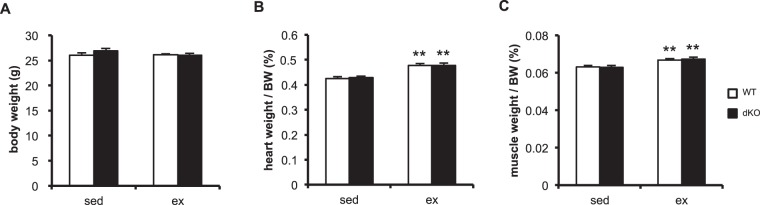
Heart and muscle weights before and after 4 weeks of voluntary wheel running. (**A**) Bodyweights of WT and KO mice. Heart (**B**) and plantaris muscle weights (**C**) per bodyweight (BW) of WT and dKO mice. Data are means ± SEM (n = 6–8). **P < 0.01 main effect of exercise.

Next, we exposed the mice to 4 weeks of voluntary wheel running to induce skeletal muscle adaptation because skeletal muscle is a highly adaptive tissue that undergoes various physiological changes in response to endurance exercise. During the experimental period, the WT and dKO mice ran similar distances (Table 3). There was no significant difference in the bodyweights of the WT and dKO mice (Fig. 4A). The voluntary running increased the heart and plantaris muscle weights (Fig. 4B,C) in both the WT and dKO mice.

**Table 3:** Running distance.

Genotype	Distance (km)
WT	11.07 ± 0.87
dKO	10.12 ± 0.63

Mean running distance per day during 4-week voluntary running. WT (n = 10), control littermate mice. dKO (n = 7), Ckmm-cre induced muscle-specific miR-23a/b clusters double-KO mice. Data are presented as means ± SEM.

**Figure 5:**
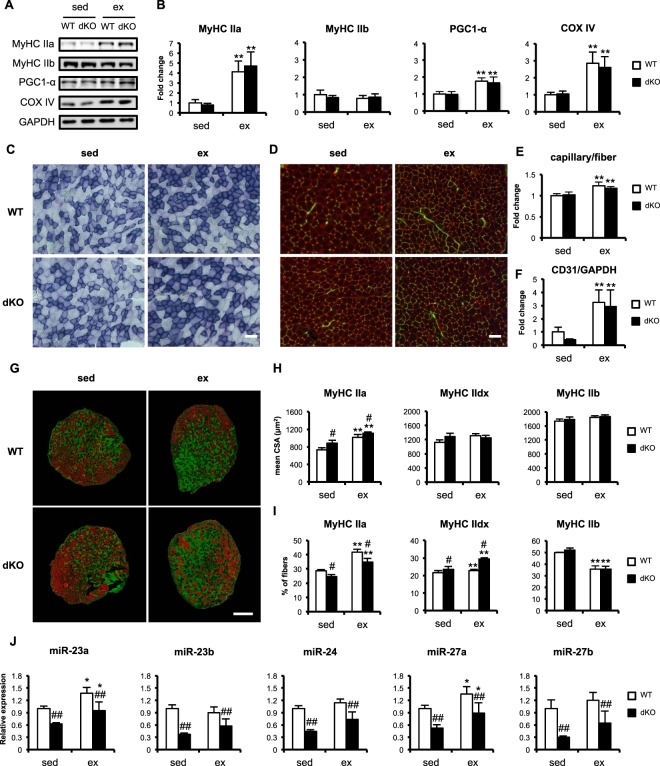
miR-23–27–24 clusters are not essential for endurance-exercise-induced muscle adaptation. (**A**) Representative images of western blotting analysis. (**B**) Quantitative data for the protein expression of MyHC type IIa, MyHC type IIb, PGC-1α, and COX IV. Data are means ± SEM (n = 6). **P < 0.01 main effect of exercise. (**C**) Representative images of SDH staining in plantaris muscle. Scale bar, 100 μm. (**D**) Representative immunofluorescent images of capillaries in plantaris muscle. Dystrophin is shown in red, CD31 in green. Scale bar, 100 μm. Quantitative data for the capillary-to-fiber ratio (**E**) and CD31 expression (**F**) in plantaris muscle. Data are means ± SEM (n = 5). **P < 0.01 main effect of exercise. (**G**) Representative images of plantaris muscle immunofluorescently stained for MyHC type IIa fibers and dystrophin in green, type IIb fibers in red. MyHC type IId/x fibers are unstained (black). Scale bar, 500 μm. (**H**) Mean cross-sectional areas (CSAs) of MyHC type IIa, MyHC type Id/x, and MyHC type IIb fibers in plantaris muscle. Data are means ± SEM (n = 4–5). ^#^P < 0.05 main effect of genotype. **P < 0.01 main effect of exercise. (**I**) Proportions of MyHC IIa, MyHC IId/x, and MyHC IIb fibers in plantaris muscle. Data are means ± SEM (n = 4). ^#^P < 0.05 main effect of genotype. **P < 0.01 main effect of exercise. (**J**) Expressions of the mature miRNAs in miR-23a/b clusters after 4 weeks of voluntary running. Data are means ± SEM (n = 5). ^##^P < 0.01 main effect of genotype. *P < 0.05 main effect of exercise. sed, sedentary group; ex, exercised group.

Generally, long-term endurance exercise increases the oxidative capacity of the skeletal muscle, represented by an increase in oxidative fibers, increased levels of mitochondrial marker proteins, such as PGC-1α and COX IV, and increased SDH activity^29–32^. Consistent with the previous studies^33–36^, MyHC IIa, PGC1-α, and COX IV increased after the 4 weeks of voluntary wheel running in both the WT and dKO mice, whereas MyHC IIb expression was not affected by either the mouse genotype or the voluntary running (Fig. 5A,B). SDH activity was also elevated by the voluntary running in both the WT and dKO mice (Fig. 5C).

We also examined whether miR-23–27–24 deletion influenced exercise-induced angiogenesis^34^. Although the capillary density and CD31 expression in the plantaris muscle was elevated after exercise training, there was no significant difference between the WT and dKO mice (Fig. 5D–F).

The 4 weeks of voluntary running also induced increases in the CSAs and numbers of glycolytic-to-oxidative fibers in the plantaris muscles (Fig. 5G). The CSA of type IIa fibers were increased by voluntary running in both the WT and dKO mice (Fig. 5H). The CSAs of type IId/x and IIb fibers were not altered by either voluntary running or the genotype (Fig. 5H). The amounts of type IIa and IId/x fibers were increased by voluntary running, whereas the amounts of type IIb fibers decreased (Fig. 5I). The muscle-specific deletion of miR-23–27–24 reduced the proportion of type IIa fibers in the plantaris muscle, but increased the proportion of type IId/x fibers, regardless of exercise (Fig. 5I).

To determine whether the voluntary running influences expression of the miR-23a/b cluster miRNAs, we quantified the post-exercise level of these miRNAs. The miR-23a and miR-27a expressions were mildly increased in both the WT and dKO mice after the 4 weeks of voluntary running. However, each miRNA in miR-23a/b clusters was still significantly reduced in the dKO mice regardless of the voluntary running (Fig. 5J,K). Because exercise-induced muscle adaptation is occurred by a consequence of cumulative bouts of exercise, we also examined whether an acute bout of exercise has an influence on expression of the miRNAs. The acute bout of wheel running decreased miR-23a and miR-24 in the WT mice. The miR-23b and miR-27a were also decreased by the acute exercise both in the WT and dKO mice while they were still significantly reduced in the dKO mice compared to WT mice (Supplemental Fig. S1). The miR-27b did not show significant changes in response to the acute exercise both in the WT and dKO mice (Supplemental Fig. S1).

Taken together, these results indicate that 4 weeks of voluntary running was sufficient to induce skeletal muscle adaptation and that the muscle-specific deletion of the miR-23–27–24 clusters did not affect those changes.

## Discussion

In this study, we have demonstrated that Ckmm-cre induced the ablation of the miR-23a/b clusters, which did not produce a significant skeletal muscle phenotype, either in the resting or exercised state. The only change observed in the dKO mice was the enlarged CSA of the oxidative muscle fibers (Fig. 3H,I). The distributions of CSA of the type IIa and type IId/x fibers in the dKO plantaris muscle were larger than in the WT (Fig. 3H), although the mean CSA only increased in the type IIA fibers (Fig. 5H). In the soleus muscle, the distributions of CSA of the type I, type IIa, and type IId/x fibers were also larger than in the WT (Fig. 3I). This suggests that the absence of the miR-23a/b clusters more strongly affected the phenotype of the oxidative fibers than that of the glycolytic fibers because the expression of the miR-23a/b clusters is greater in the soleus (where slow-twitch and oxidative fibers are dominant) than in the plantaris (where fast-twitch and glycolytic fibers are dominant)^16,27^.

However, the muscle weights did not increase in the dKO mice despite the increase in CSAs (Table 1). The enlargement of the type IIa fibers might not have been sufficient to increase the total plantaris weight, because the predominant fiber type in this muscle is MyHC type IIb, the CSA of which was not altered in the dKO mice. The reduced percentage of type IIa fibers in the dKO plantaris muscle might also have offset the increased CSA, so that the total proportion of type IIa fibers did not differ in the WT and dKO plantaris muscles. However, we observed no significant changes in the muscle weight, proportions of fiber types, or total fiber numbers in the dKO soleus muscle, despite the increased CSA (Fig. 3F,G). It can be assumed that the decreasing trend in the proportion of type IIa fibers, which is the dominant fiber type in the soleus (Fig. 3F, P = 0.244) and in the total fiber numbers (Fig. 3G, P = 0.28), although it was not statistically significant, may have, at least partly, cancelled out the CSA-enlargement-induced increase in muscle mass. The skeletal muscle strength was unchanged in the dKO mice (Table 2), suggesting that the changes in the oxidative fibers caused by the ablation of the miR-23a/b clusters did not induce functional changes in the skeletal muscle.

We have previously reported that miR-23 suppresses PGC-1α by binding to the 3′ UTR of its transcript^37^. PGC-1α is a critical transcriptional coactivator that regulates exercise-induced skeletal muscle adaptation, including mitochondrial biogenesis^38^, energy metabolism^32^, and angiogenesis^34^. A negative correlation between miR-23 and PGC-1α expression in response to an acute bout of endurance exercise has also been reported^24^. In this study, we demonstrated that PGC-1α, oxidative MyHC type IIa, and COX IV expression and SDH activity in the skeletal muscle were unchanged in the dKO mice (Fig. 3B,D), even in response to voluntary running (Fig. 5B,C). This is consistent with our previous study, in which miR-23a transgenic mice did not differ from the WT in response to exercise^39^. In the dKO mice, miR-23a and miR-27a were elevated after the 4 weeks of voluntary running although their expression levels were still lower than those in the WT mice (Fig. 5J). It might be possible that the elevated expressions of miR-23a and miR-27a compensate the exercise-induced muscle adaptation in the dKO mice. Taken together, our results suggest that the miR-23a/b clusters are not essential for endurance-exercise-induced muscle adaptation.

Angiogenic adaptation in response to exercise is important because it allows the management of the increased the expenditure of energy and oxygen that occurs in skeletal muscle. It has been reported that skeletal-muscle-derived angiogenic signals, such as vascular endothelial growth factor (VEGF), are important in exercise-induced angiogenesis^40^. Previous studies have demonstrated that miR-23 and miR-27 exert proangiogenic effects by targeting sprouty 2 and semaphorin 6A, which inhibit angiogenesis^41^, whereas miR-24 promotes endothelial cell apoptosis and inhibits vascularization by targeting the endothelial transcription factor GATA2 and the P21-activated kinase PAK4, which inhibits proapoptotic signals in endothelial cells^42^. In this study, we have demonstrated that the deletion of the miR-23a/b clusters in skeletal muscle did not inhibit the increase in capillary formation in response to endurance exercise (Fig. 5D–F). This result suggests that muscle miR-23a/b clusters are not essential for exercise-induced angiogenesis. Because each miRNA expressed from the miR-23a/b clusters has either a pro- or antiangiogenic function, the overall angiogenic effects of the clusters might be neutral.

We generated muscle-specific miR-23a/b-cluster-dKO mice using the Ckmm-cre mouse. The Ckmm-cre mouse has been used in a number of studies of gene functions in skeletal muscle^26,43^. Muscle creatinine kinase promoter is activated in the postdifferentiated stage of skeletal muscle^26^, which first emerges on embryonic day 13.5^44^ when multinucleated primary myofibers are generated^35,45^. Also, it has been reported that Ckmm-cre is activated in entire myonuclei within myofibers at 1.5 months of age when myonuclear accretion is completed for mice muscle growth^26^. Therefore, the phenotypes of the dKO mice are attributable to the absence of the miR-23a/b clusters in multinucleated skeletal muscle.

Each miRNA expressed from the miR-23a/b clusters in the dKO was significantly reduced in the skeletal muscle, but we still detected some residual miRNA expression (Fig. 2D,E). Because mononucleated muscle stem cells (also called ‘satellite cells’) are resident in the adult skeletal muscle and other cell types, such as endothelial cells, neuronal cells, and fibroblasts, which are not affected by Ckmm-cre (Fig. 2C)^43^, it can be assumed that the miRNAs expressed from the miR-23a/b clusters in these cells might be present in the dKO mice. We also noted that there were more residual miR-23a cluster miRNAs than miRNAs from the miR-23b cluster in the dKO skeletal muscles (Fig. 2B,C), whereas the miRNAs expressed from the miR-23a cluster were more reduced in the lungs of endothelial-cell-specific miR-23a/b-cluster dKO mice^46^. Although the transcriptional regulation of the miR-23a/b clusters has not been clarified, it has been reported that the promoter regions of the miR-23a cluster and miR-23b cluster are differently activated by MEF-2C and PGC-1α^27^, and that the promoter activity of the miR-23a cluster may differ in fibroblasts and myocytes^47^. The different transcriptional regulation of the miR-23a/b clusters in each cell type and the different transcriptional regulation of the miR-23a and miR-23b clusters may contribute to the different patterns of the residual miRNAs expressed from the miR-23a/b clusters in the dKO mice tissues.

Because the clustered miRNAs are simultaneously transcribed in the same polycistronic transcript^48,49^ and have targets that are involved in similar biological processes^4,50^, it can be assumed that miR-23, miR-27, and miR-24 function coordinately in response to physiological changes. Several studies have reported that miR-23^51,52^, miR-27^36^, and miR-24^53^ are involved in myoblast differentiation. Most of these studies reported that miR-23, miR-27, and miR-24 promote myoblast differentiation^36,52,53^, whereas the other study reported that miR-23a exerts the opposite effect^51^. Wang *et al*. reported that miR-23a is downregulated in the early stages of myogenesis and upregulated dramatically in the late stages to inhibit muscle differentiation by targeting the muscle structural proteins MYH1, MYH2, and MYH4^51^. In contrast, Mercatelli *et al*. reported that both miR-23a and miR-23b are upregulated gradually from the early stages of myoblast differentiation, to promote myoblast differentiation by targeting the expression of thioredoxin reductase 1 (TRXR1). TRXR1 is part of the thioredoxin system, which controls redox homeostasis but also appears to repress the myogenic genes encoding myogenin and MyHC in the later stages myoblast differentiation^52^. miR-27 is upregulated in the early stages of myoblast differentiation and promotes myoblast differentiation by inhibiting paired-box transcription factor 3 (PAX3), which maintains the undifferentiated state^36^. miR-24 expression is upregulated gradually during myoblast differentiation^53^, and promotes myoblast differentiation by increasing the expression of both the early and late myogenic genes, MEF2, myogenin, and MyHC^53^. These findings indicate that individual miRNAs from the miR-23a/b clusters regulate myoblast differentiation by controlling the expression of myogenic genes. Because the Ckmm-cre-induced miR-23a/b-cluster dKO mice displayed no significant abnormalities in their skeletal muscle, it is conceivable that the functions of the miR-23a/b clusters more strongly affect myoblasts than fully differentiated muscle fibers. Further genetic studies, especially targeting miR-23a/b clusters in the early stage of muscle development, should demonstrate the precise roles of the miR-23a/b clusters in skeletal muscle biology.

## Materials and Methods

### Generation of mice with muscle-specific knockout of the miR-23~27~24 clusters

The plasmid pDT-loxP-loxPFRT-PGKneo-loxPFRT was kindly supplied by S. Takahashi (University of Tsukuba) and used to construct vectors targeting the miR-23a and miR-23b clusters (Fig. 1A). Each 5′ arm (4.2 kb), central domain (containing the miR-23a or miR-23b cluster, 1.5 kb), and 3′ arm (2.1 kb) of the miR-23a and miR-23b clusters were amplified from mouse genomic DNA and ligated into the targeting vector. The vector was then linearized and introduced into mouse embryonic stem (ES) cells derived from C57BL6/J mice by electroporation. The transfected ES cells were selected with Geneticin^®^ (G418, GIBCO), and the homologously recombined cells were identified with a Southern blotting analysis using 5′ and 3′ external probes (Fig. 1A,B). The properly transfected ES clones were injected into blastocysts and transferred to pseudopregnant female mice to generate chimeric progeny mice. The chimeras were intercrossed to facilitate the germline transmission of the targeted allele, and then crossed with CAG-FLPe mice (Riken BRC, Ibaraki, Japan) to remove the neomycin-resistance cassette with flippase–flippase recognition target (Flp–*FRT*) recombination.

Muscle-specific miR-23a/b-cluster-knockout mice were generated by cross-breeding miR-23a/b-cluster-double-floxed mice and Ckmm-cre mice (Jackson Laboratory). The tail DNA of the mice was genotyped with PCR with the following primer pairs: miR-23a–27a–24-2, 5′-GCT CCA ACC TTC CTA CGG ATC GAT GC-3′ and 5′-CCT GAG GGG ACA TAA CTG GCT TT-3′; miR-23b–27b–24-1, 5′-TGC CCC CTG AGT GAG CAA ATC C-3′ and 5′-TCT ACA GAC AAG GCC CTT CAG ACA G-3′. The primers were also used to examine Cre-induced recombination. PCR product size for WT, floxed and KO alleles of miR-23a cluster are 1001 bp, 1107 bp and 197 bp, respectively, and those of miR-23b cluster are 1535 bp, 1670 bp and 170 bp, respectively. The Ckmm-cre mice were identified with PCR using the specific primers: 5′-ATC AGC TAC ACC AGA GAC GGA AA-3′ and 5′-GAG GTT CAC AGG GGG AGA AC-3′.

### Animal experiments

All the mice were cage-housed in a temperature-controlled (21 °C) environment under a 12 h light/12 h dark cycle, with free access to food and water according to the Guideline for Experimental Animal Care issued by the Prime Minister’s Office of Japan. Eighteen-week-old male Ckmm-cre;miR-23a/b-cluster-double-floxed mice and their miR-23a/b-cluster-double-floxed littermates were used for the experiments. The mice in the exercise group were housed individually in cages equipped with running wheels^54^. They were familiarized with the cages for 2 days before commencing voluntary wheel running. The wheel rotations were counted with a computer system (Meltiest, Toyama, Japan) and the total running distance was calculated. The sedentary mice were also housed individually in the same-sized cages without running wheels during the experimental period. For long-term (4 weeks) voluntary running mice (22 weeks old of age), the muscle samples were harvested following a 24-hour resting period (with locked wheels) after the last bout of running. An acute bout of wheel running exercise was conducted as described previously^55–57^, and the mice were sacrificed 3 hours after the final session. Their skeletal muscles were harvested in the appropriate buffer or embedded in OCT compound. The animal protocols were approved by the Animal Care and Use Committee of the University of Tokyo and Waseda University.

### Muscle strength

The grip strength of the mice in all four limbs was measured as described previously^58,59^. Briefly, each mouse was placed on the grid that attached to a force measurement device (DST-50N, Imada Co., Ltd) and pulled back horizontally until its grip failed. The procedure was conducted three times in succession as a set, and the strength in each session was recorded. Each mouse was subjected to a total of three sets, on 3 consecutive days. The mean strength of each mouse was adjusted to its bodyweight.

### Muscle fiber isolation

The isolation of muscle fiber was performed as described previously^60,61^. Breifly, extensor digitorum longus (EDL) and flexor digitorum brevis (FDB) muscles were harvested and incubated with 0.2% collagenase II (Worthington Biochemical Corporation) in DMEM for 40 minutes. Isolated muscle fibers are collected with glass pipette.

### RT-PCR

Total RNA was extracted from the animal tissues with Isogen® II (FujiFilm Wako Pure Chemical Corporation, Japan), according to the manufacturer’s instructions. 1 μg of total RNA was reverse transcribed using ReverTra Ace qPCR RT kit (Toyobo, Osaka, Japan). Ex Taq HS (Takara, Osaka, Japan) was used for PCR with the following gene-specific primers: Cre, 5′-GCC TGC ATT ACC GGT CGA TGC-3′ and 5′-CAG GGT GTT ATA AGC AAT CCC-3′; Gapdh, 5′-GAC CCC TTC ATT GAC CTC AAC-3′ and 5′-TAA GCA GTT GGT GGT GCA GGA-3′. Thunderbird SYBR qPCR Mix (Toyobo, Osaka, Japan) was used for real-time PCR with the following gene-specific primers: Myostatin, 5′-ACA GAG TCT GAC TTT CTA ATG CAA G-3′ and 5′-GGA GTC TTG ACG GGT CTG AG-3′; MuRF1, 5′-CTT CCA AGG ACA GAA GAC TGA GC-3′ and 5′-AGT CCT CCA GCT GAG AGA TGA T-3′; Atrogin1, 5′-CTC AGA GAG GCA GAT TCG CAA-3′ and 5′-AGG GTG ACC CCA TAC TGC T-3′; Gapdh, 5′-AAA TGG TGA AGG TCG GTG TG-3′ and 5′-TGA AGG GGT CGT TGA TGG-3′. Gapdh was used as internal standards.

### miRNA analysis

TaqMan™ MicroRNA Assays (Applied Biosystems, Foster City, CA) for miR-23a (no. 000399), miR-23b (no. 000400), miR-27a (no. 000408), miR-27b (no. 000409), and miR-24 (no. 000402) were used to analyze the miRNAs, according to the manufacturer’s instructions. Briefly, 10 ng of total RNA was combined with reverse transcription (RT) reaction mixture containing RT primers, reverse transcription buffer, dNTPs with dTTP, reverse transcriptase, and RNase inhibitor, and the RT mixture was incubated with the following thermal protocol: 30 min at 16 °C, 30 min at 42 °C, and 5 min at 85 °C. Real-time PCR for each miRNA was performed with the Applied Biosystems StepOne Real-time PCR System in 96-well plates.

### Western blotting analysis

The dissected muscle tissues were immediately transferred to complete protein-loading buffer and homogenized with a glass homogenizer. The complete protein-loading buffer contained 50 mM Tris-HCl (pH 6.8), 1% SDS, 10% glycerol, 20 mM dithiothrietol, 127 mM 2-mercaptoethanol, 0.01% bromophenol blue, protease inhibitors (Roche), and phosphatase inhibitors (Sigma-Aldrich). The muscle homogenates were transferred to 1.5 ml microfuge tubes, denatured for 5 min at 95 °C, and centrifuged for 10 min at 12,000 × g. The protein concentrations of the homogenates were measured with the RC DC™ Protein Assay kit (Bio-Rad). The total proteins (10–40 μg) were loaded onto 6%, 7.5%, 10% and 12% gels and separated with SDS-PAGE for 1.5–2 h at 100 V. After SDS-PAGE, the proteins were transferred to a nitrocellulose membrane (Hybond ECL, GE Healthcare) at 100 V for 1 h in a chamber filled with transfer buffer. Nonspecific protein binding was blocked with 5% skim milk in Tris-buffered saline (TBS) containing 0.05% Tween 20 (TBST) for 1 h at room temperature. The membranes were incubated with the following primary antibodies: anti-myosin heavy chain (MyHC) type I (BA-F8, DSMZ, 1:100), anti-MyHC type IIa (SC-71, DSMZ, 1:100), anti-MyHC type IIb (BF-F3, DSMZ, 1:100), anti-PGC1-α (AB3242, Millipore, 1:500), anti-cytochrome c oxidase complex IV (anti-COX IV; #4844, Cell Signaling Technology, 1:1000), anti-CD31 (anti-PECAM-1; sc-46694, Santa Cruz Biotechnology, INC., 1:400), anti-Phospho-Akt (#4060, Cell signaling Technology, 1:1000), anti-Akt (#9272, Cell signaling Technology, 1:1000) and anti-glyceraldehyde 3-phosphate dehydrogenase (anti-GAPDH; MAB374, Millipore, 1:1000). ECL™ Rabbit IgG, HRP-linked F(ab’)_2_ fragment (GE Healthcare) and Goat Anti-Mouse IgG (H + L)–HRP Conjugate (Bio-Rad) were used as the secondary antibodies. The signals were immunodetected with Amersham ECL Prime Western Blotting Detection Reagent (GE Healthcare) using the LAS-3000 Imaging System (Fuji Film, Japan). The signals were quantified with the ImageJ software (National Institutes of Health, Bethesda, MD).

### Immunofluorescent staining

The OCT-embedded muscles were cut transversely into 10 μm sections at −20 °C and collected immediately on glass slides. The sections were fixed with 4% paraformaldehyde in phosphate-buffered saline (PBS) on ice and permeabilized with 0.3% Triton X-100 in PBS. Monoclonal antibodies directed against MyHC I (BA.F8), MyHC IIa (SC.71), MyHC IIb (BF.F3), and CD31 (MCA2388, AbD Serotec) were diluted 1:25, 1:250, 1:25, and 1:100, respectively, in 5% normal goat serum (NGS) in PBS and used as the primary antibodies to identify the muscle fiber types and capillaries. Mouse anti-dystrophin antibody (D8043, Sigma) was diluted 1:100 in 5% NGS/PBS and used to determine the shapes of the myofibers. Alexa-Fluor-405-conjugated goat anti-mouse IgG2b, Alexa-Fluor-488-conjugated goat anti-mouse IgG1, Alexa-Fluor-549-conjugated goat anti-mouse IgM, and Dy549-conjugated goat anti-mouse IgG1 antibodies were all diluted 1:100 in 5% NGS/PBS and used as the secondary antibodies. All the secondary antibodies and NGS were purchased from Jackson ImmunoResearch Laboratories (PA, USA). Images of the immunofluorescently stained cross-sections were taken with an IX-70 fluorescence microscope (Olympus, Japan) equipped with a DS-Ri1 digital camera (Nikon, Japan). The subjects were blinded to investigators when the images were analyzed. The cross-sectional areas (CSAs) and capillary densities were calculated with the ImageJ software.

### Enzymatic staining

The transverse sections of the OCT-embedded muscles were stained for succinate dehydrogenase (SDH), as described previously^62^. Images of the SDH-stained cross-sections were taken with an IX-70 fluorescence microscope equipped with a DS-Ri1 digital camera.

### Statistics

All values are presented as means ± standard error of mean (SEM). Comparisons for two factors; genotype (WT vs dKO) and exercise (sed vs ex) were made with two-way factorial analysis of variance (ANOVA). Tukey’s HSD test was performed as post-hoc test. Student’s *t* test was used for comparisons of two groups. All statistical analyses were performed with IBM SPSS Statistics 24 (IBM, USA).

## Supplementary information

Supplementary Fig.1
