# Supplementary material for: Loss of microRNA-23–27–24 clusters in skeletal muscle is not influential in skeletal muscle development and exercise-induced muscle adaptation
Source: Sci Rep. 2019 Jan 31;9:1092. doi: 10.1038/s41598-018-37765-3 (PMC6355808; doi:10.1038/s41598-018-37765-3)
Supplement: Supplementary file 1 — Supplementary Fig.1 [file 41598_2018_37765_MOESM1_ESM.pdf]

Supplementary information

Loss of microRNA-23–27–24 clusters in skeletal muscle is not influential in skeletal muscle development and exercise-induced muscle adaptation.

Minjung Lee[1*], Shogo Wada[1*], Satoshi Oikawa[2], Katsuhiko Suzuki[3], Takashi Ushida[1], Takayuki Akimoto[1,3]

[1] Division of Regenerative Medical Engineering, Center for Disease Biology and Integrative Medicine, Graduate School of Medicine, The University of Tokyo, Tokyo 113-0033, Japan
[2] Graduate School of Comprehensive Human Science, University of Tsukuba, Ibaraki 305-8571, Japan
[3] Faculty of Sport Sciences, Waseda University, Saitama 359-1192, Japan

The current affiliation of S.W. is the Department of Medicine and Cardiovascular Institute, Perelman School of Medicine, University of Pennsylvania, Philadelphia, PA, USA.
*: equal contributions

Correspondence and requests for materials should be addressed to T.A. (e-mail: axi@waseda.jp)

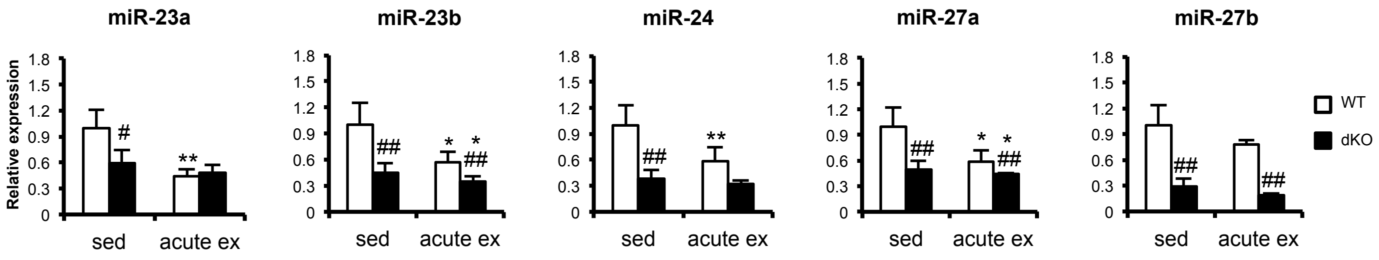

Supplementary Figure S1. Mature miRNAs in miR-23a/b clusters in skeletal muscle after an acute bout of wheel running.

WT, wildtype. dKO, miR-23a/b clusters double KO. sed, sedentary group. acute ex, an acute bout of wheel running group.

$^{\#}$P < 0.05 and $^{\#\#}$P < 0.01 compared with WT. *P < 0.05 and **P < 0.01 compared with sed. Data are means ± SE (n = 3–5).

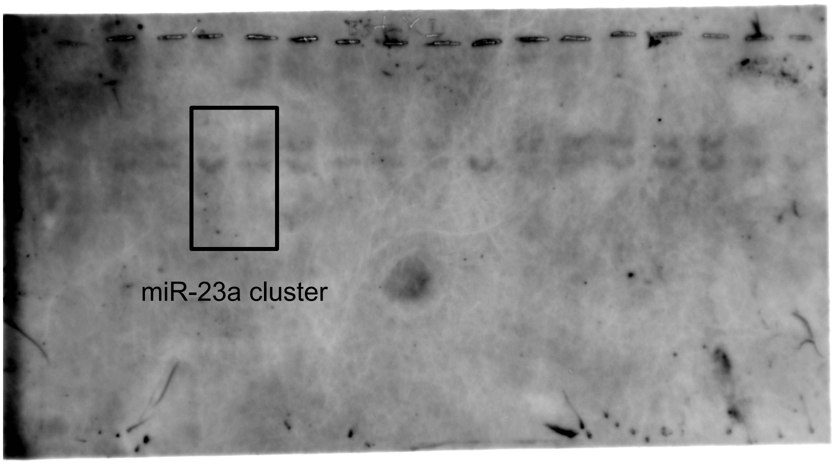

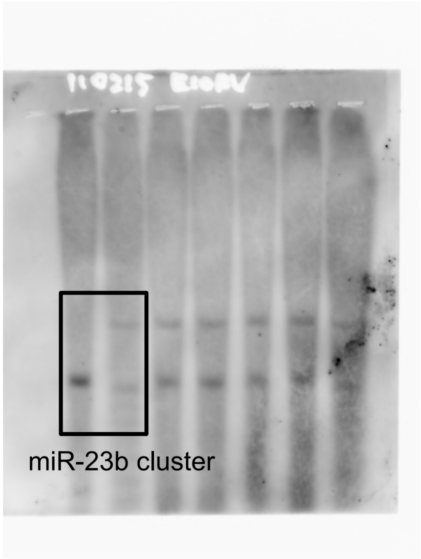

Supplementary Figure S2. Representative blot images displayed in Fig. 1B

Fig. 2A

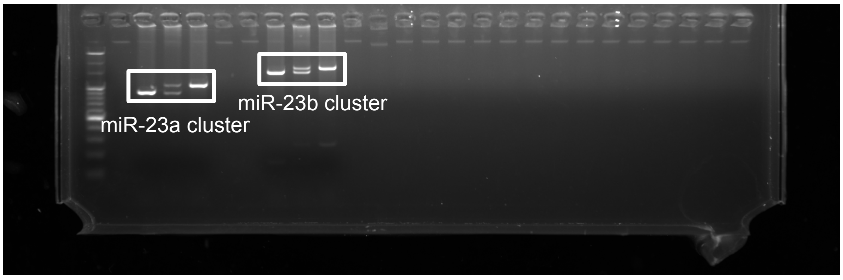

Fig. 2B

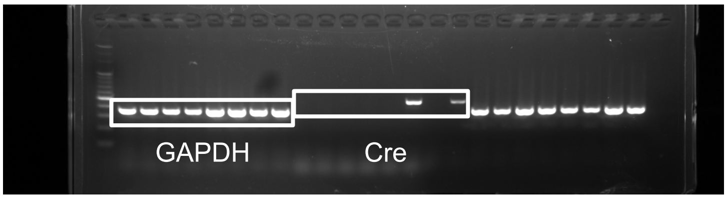

Fig. 2C

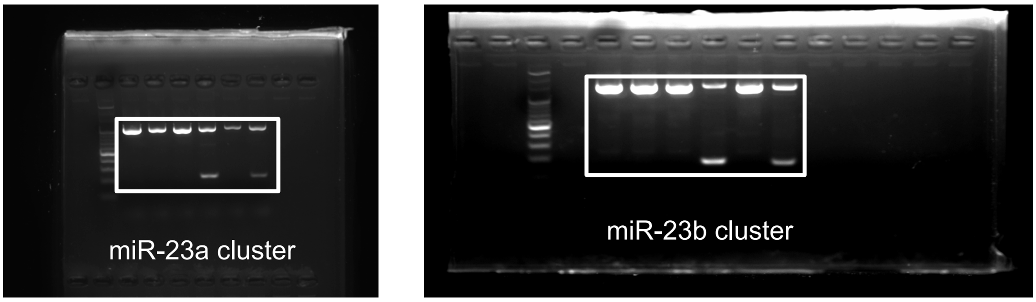

Supplementary Figure S3. Representative gel images displayed in Fig. 2

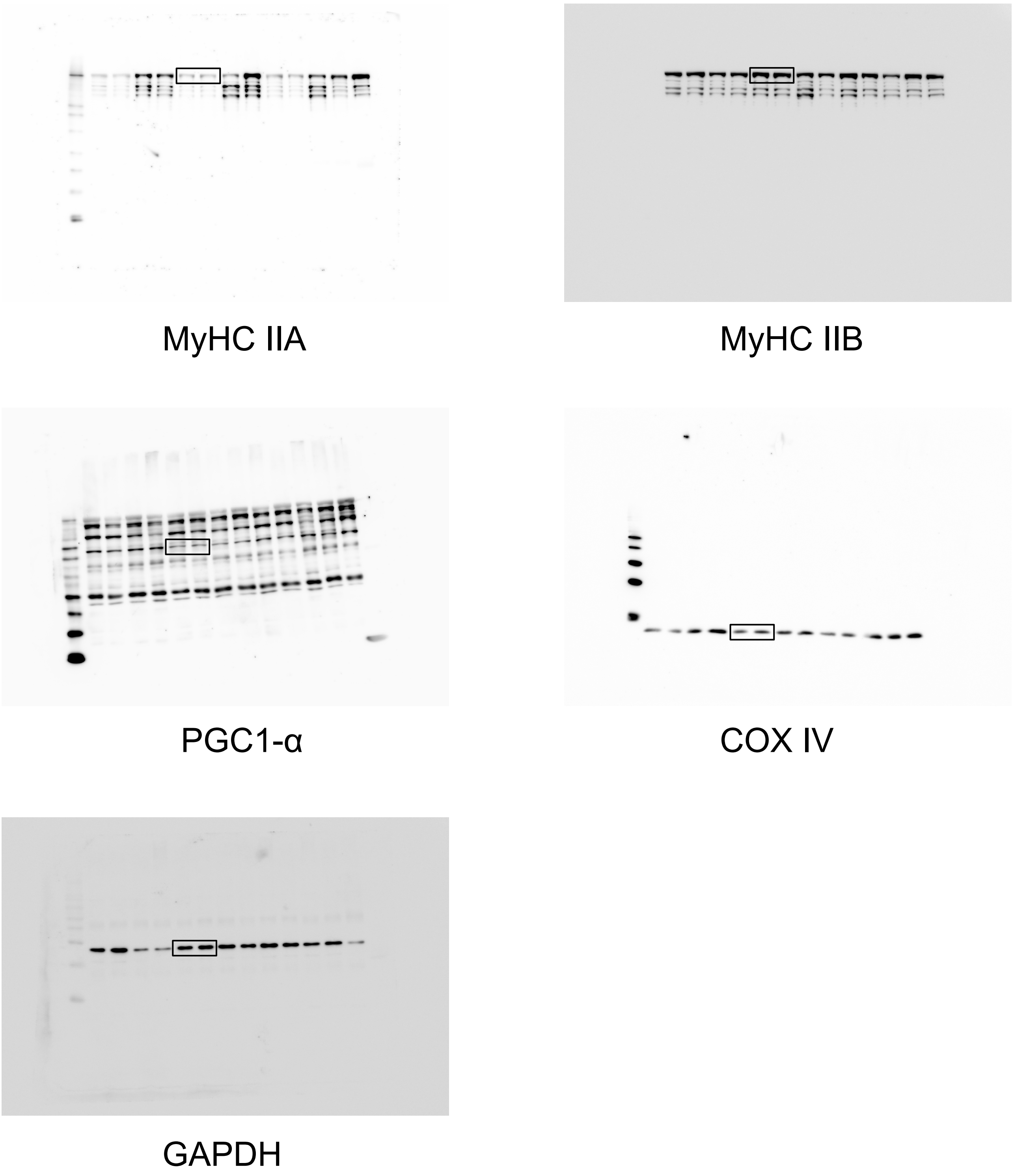

Supplementary Figure S4. Representative blot images displayed in Fig. 3A

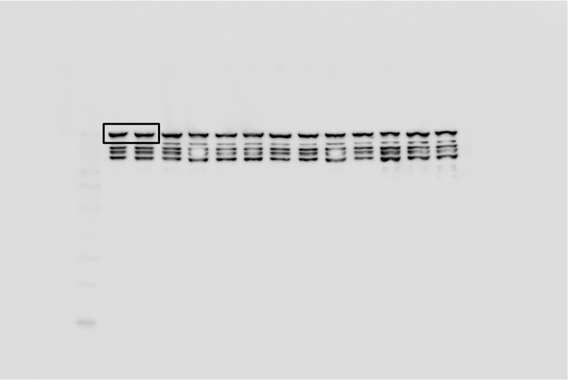

MyHC I

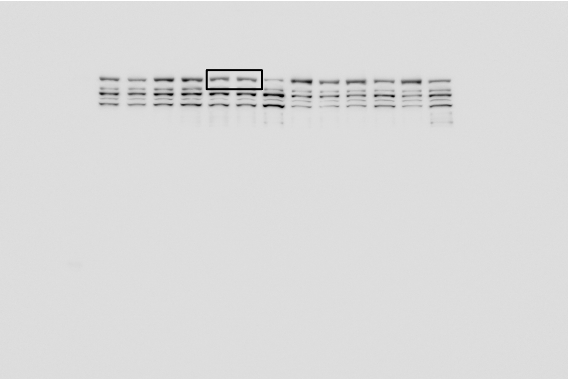

MyHC IIA

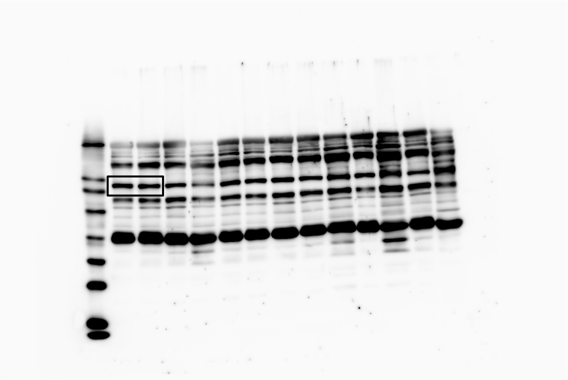

PGC1-α

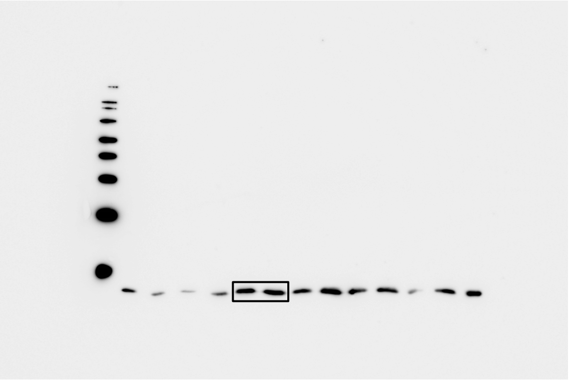

COX IV

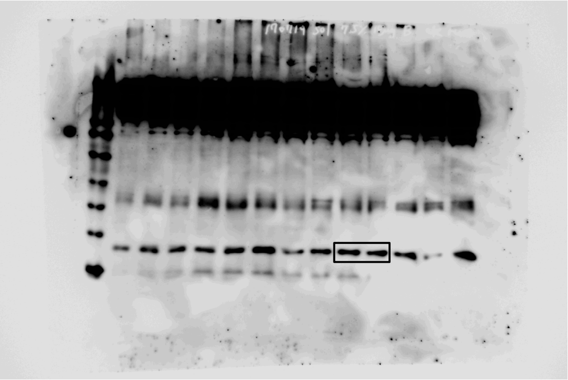

GAPDH

Supplementary Figure S5. Representative blot images displayed in Fig. 3C

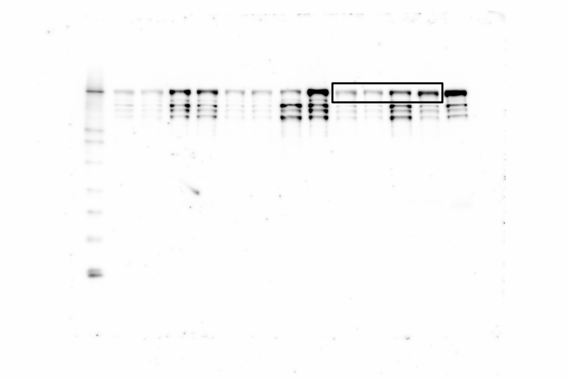

MyHC IIA

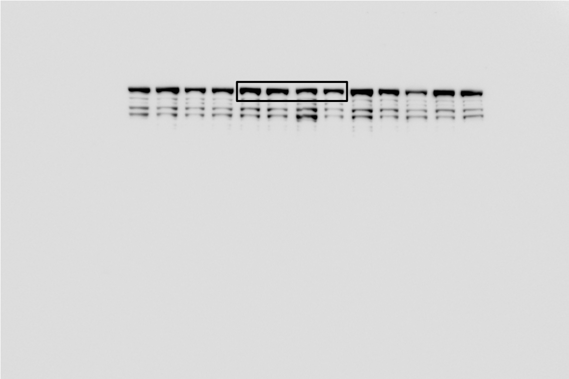

MyHC IIB

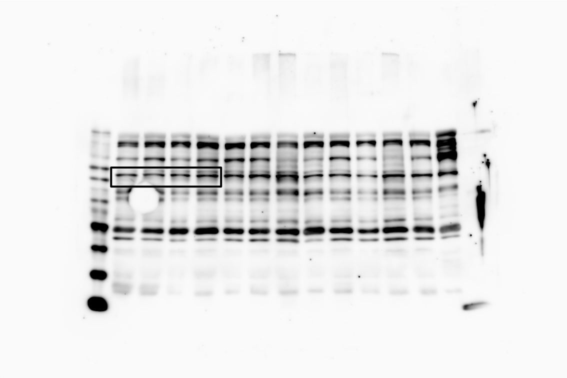

PGC1-α

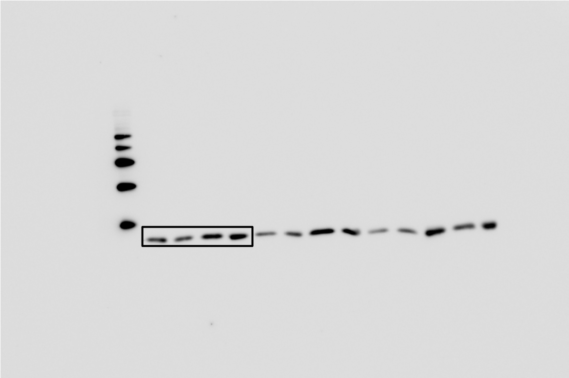

COX IV

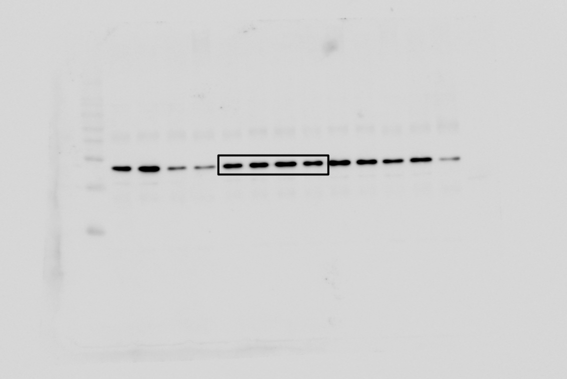

GAPDH

Supplementary Figure S6. Representative blot images displayed in Fig. 5A
